# *Capnocytophaga canimorsus* Septicemia With Sepsis-Induced Coagulopathy and Endocarditis

**DOI:** 10.1155/2024/4010115

**Published:** 2024-09-18

**Authors:** Jeannine L. Kühnle, Maximilian Leitner, Vitalie Mazuru, Kai Borchardt, Sören L. Becker, Franziska Roth, Robert Bals, Philipp M. Lepper, Hans-Joachim Schäfers, Isabella T. Jaumann

**Affiliations:** ^1^ Department of Internal Medicine V-Pneumology, Allergology, and Intensive Care Medicine University Hospital and University of Saarland, Homburg, Germany; ^2^ Institute of Medical Microbiology and Hygiene Saarland University, Homburg, Germany; ^3^ Department of Acute and Emergency Medicine University Hospital and University of Saarland, Homburg, Germany; ^4^ Department of Thoracic and Cardiovascular Surgery University Hospital and University of Saarland, Homburg, Germany

**Keywords:** *Capnocytophaga canimorsus*, disseminated intravascular coagulation, sepsis, sepsis-induced coagulopathy, thrombotic microangiopathy

## Abstract

*Capnocytophaga canimorsus* is a rare cause of serious infections with a high mortality of 10% to 30%. It is usually found in the oral cavity of cats and dogs and can cause severe sepsis in immunocompromised patients. An 81-year-old female Caucasian patient presented with *C. canimorsus* sepsis after a dog bite in her finger three days before presentation to our emergency department. She initially was presented to us with sepsis, thrombopenia, and schistocytes in her laboratory findings, suggesting the differential diagnoses of the multiple subtypes of thrombotic microangiopathy. She was admitted to the medical intensive care unit of the University Hospital of Saarland because of septic shock with circulatory insufficiency. The patient received plasmapheresis, antibiotics, and dialysis, under which she improved significantly. The fingertip of the affected finger developed necrosis and had to be amputated. Furthermore, the patient was diagnosed with a mitral valve endocarditis, a very rare complication of *C. canimorsus* infection. It was treated conservatively with antibiotics and was no longer detectable 8 weeks after the diagnosis. Surgical intervention was not needed. The case describes well that it is still difficult to distinguish between thrombotic thrombocytopenic purpura (TTP), disseminated intravascular coagulation (DIC), and sepsis-induced coagulopathy (SIC), especially in the early phases of acute disease, especially in *C. canimorsus-*induced sepsis.

## 1. Introduction

*Capnocytophaga canimorsus* is a rare cause of serious infections with a high mortality of 10% to 30% [1]. Here, we describe the case of a patient with *C. canimorsus* sepsis. She initially presented with thrombopenia and schistocytes in her laboratory findings, suggesting the differential diagnoses of the multiple subtypes of thrombotic microangiopathy. Furthermore, the patient presented with a mitral valve endocarditis, a very rare complication of *C. canimorsus* infection.

## 2. Case History

An 81-year-old female Caucasian patient presented to the emergency department with fever, dizziness, and weakness during the past 2 days. She complained of nausea and vomiting prior to admission. She reported that her dog (a dachshund) had bitten her in the finger three days prior to admission, and she had not seen a doctor on the day of the accident. The following day, she started feeling weak.

She had a history of coronary heart disease, and a bare metal stent had been implanted in her right coronary artery in 2011. Since then, she had been taking prasugrel 10 mg once a day. She also had a history of hypertension treated with 10 mg amlodipine twice daily, 5 mg ramipril twice a day, and metoprolol 47.5 mg once a day. Six months earlier, she had been treated for a peritonsillar abscess in our hospital. She did not take any other medications and had not been to any foreign countries in the last months.

On initial examination through the rescue service, she had a Glasgow Coma Scale (GCS) of 15 (opened her eyes spontaneously, was oriented to time, person, and place, and obeyed motoric commands). Her systolic blood pressure was 60 mmHg, heart rate 130/min, respiratory rate 29/min, and oxygen saturation 79% on room air. The temperature was 39.7°C. 1.500 mL crystalloid, 200 mg cafedrine hydrochloride/10 mg theodrenaline hydrochloride, and 8 mg ondansetron were administered.

**Figure 1:**
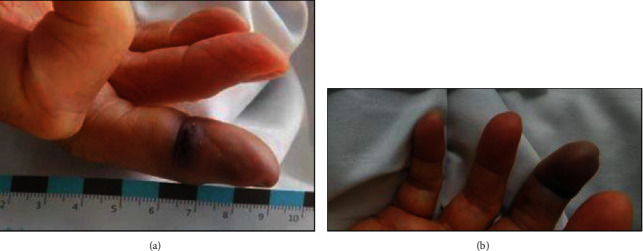
(a) Finger D3 of the right hand at admission and (b) finger D3 of the right hand one day after admission.

**Figure 2:**
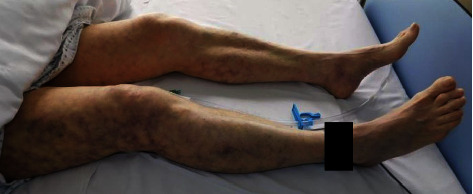
Mottling of the patient's legs on admission (mottling score 4).

On examination in the emergency department, the patient was tired but conscious with a GCS of 15. The temperature was 39.9°C, the blood pressure was 107/57 mmHg, the pulse was 110/min, and the oxygen saturation was 90% with 12 L of oxygen per reservoir mask. The heart sounds were normal. The fingertip of D3 on her right hand (Figure 1(a)) was livid with a wound (1 cm × 0.3 cm) between the first and the second phalanx. Both of her legs (Figure 2) showed profound mottling up to the groin with a mottling score of four points [2].

Laboratory investigations showed an elevated C-reactive protein (CRP 186.2 mg/L) without leukocytosis. Creatinine levels were elevated (1.41 mg/dL), and the platelets were mildly reduced (114 × 10^9^/L) (Table 1). An arterial blood gas (ABG) was acquired which showed an elevated lactate (6.5 mmol/L). Additional crystalloid fluids were given, and anti-infective treatment with piperacillin/tazobactam was started for sepsis with an initial SOFA score of six points. A chest X-ray was performed, which showed no abnormalities except for basal dystelectasis. The urine culture was negative for bacteria. Trauma surgery evaluated the patient's finger on a consultative basis and saw the need for surgical treatment. The tetanus immunization of the patient was complete. An arterial and a central line were placed, and three pairs of blood cultures were sent to our microbiology department. The patient was then transferred to our intensive care unit.

Echocardiography showed a reduced left ventricular ejection fraction of 40%, and dobutamine (0.0056 *μ*g/kg/min) was started. Until the next day, the patient received 3.5 L of crystalloid volume resuscitation but needed vasopressor support with noradrenaline (max. 0.14 µm/kg/min). She developed anuric acute renal failure.

The laboratory data on the next day showed bicytopenia with anemia (hemoglobin 9.9 g/dL) and thrombopenia (24 × 10^9^/L) as well as leukocytosis (21.1 × 10^9^/L). Total bilirubin was elevated (3.3 mg/dL). Haptoglobin (74 mg/dL) was normal. The inflammatory parameters were elevated (CRP 259.4 mg/L and procalcitonin 118 ng/mL).

Laboratory data were repeated 12 h later and showed a worsening thrombopenia (14 × 10^9^/L) with normal but declining levels of haptoglobin (37 mg/dL).

Because of her anuric kidney failure, we placed a dialysis catheter as well as a new central line and removed the one which was placed in the emergency room. She had relevant bleeding of the removal site, and one platelet concentrate was substituted.

Regarding the differential diagnosis of disseminated intravascular coagulation (DIC), the patient scored only three points in the ISTH DIC score, which consists of the patient's platelet count, international normalized ratio, fibrinogen, and fibrin D-dimer [3]. Therefore, DIC was considered but not the most likely diagnosis.

Other possible differential diagnoses in this case included thrombotic thrombocytopenic purpura (TTP) and hemolytic uremic syndrome (HUS), which both present with thrombopenia and hemolysis. HUS is most common following infection with Shiga toxin–producing *Escherichia coli* and typically accompanied by bloody diarrhea, which was missing in our case [4]. Pathophysiologically, patients with TTP develop thrombi composed of platelets and von Willebrand factor (vWF) caused by deficiency in the activity of plasma enzyme ADAMTS13. ADAMTS13 is a zinc-containing metalloproteinease enzyme that cleaves vWF into smaller sizes. If the activity is impaired, the large vWF multimers accumulate on the endothelial surface. This leads to accumulation of platelets as well as possible thrombus formation. For the diagnosis, ADAMTS13 activity must be less than 10% [5].

All forms of HUS affect the capillary area of the kidney through complement-mediated endothelial cell injury [6]. A small proportion of patients with HUS do not present with bloody diarrhea, and in this case, the diagnosis of atypical hemolytic uremic syndrome (aHUS) can be suspected. To confirm the diagnosis, specific findings in the renal biopsy are crucial [7].

On the third day, laboratory data showed a further decrease in the platelet count (39 × 10^9^/L) with rising bilirubin levels (total bilirubin 3.5 mg/dL and direct bilirubin 1.28 mg/dL). Haptoglobin was < 5 mg/dL. There were 20 per mille fragmentocytes in the peripheral blood smear, and LDH was elevated at 1.083 U/L. These findings were clinically suggestive of TTP, and plasmapheresis with 2.5 L of fresh frozen plasma was started once per day. Her PLASMIC score showed intermediate risk for severe ADAMTS13 deficiency with five points. Prednisolone (1 mg/kg) was started.

The patient received unfractioned heparin in a prophylactic dose (500 IE/h). Because of worsening thrombocytopenia, HIT ELISA test was performed and was negative. The Coombs test came back normal. To confirm the diagnosis of TTP, levels of ADAMTS13 antigen and ADAMTS13 antibodies were sent on Day 3 before starting plasmapheresis and came back normal, and TTP was ruled out. ADAMTS13 activity was reduced at 47% (range 60%–121%). Plasmapheresis was stopped.

C3c was reduced with 78.9 mg/dL, and the level of C4 and Factor I was normal. Factor H was also reduced with 244 *μ*g/mL. From Day 4 onward, platelet counts showed increasing trends. Blood cultures were positive for Gram-negative rods after 11 h of incubation, which could be specified as *C. canimorsus* using direct matrix–assisted laser desorption/ionization time-of-flight mass spectrometry on the positive blood culture fluid. This identification was later confirmed by culture growth on agar plates, but resistance testing was only successful after a prolonged incubation of 11 days on specific agar media. The pathogen was susceptible to ampicillin–sulbactam, piperacillin–tazobactam, ceftriaxone, meropenem, and ciprofloxacin.

Four days after admission, the patient was hemodynamically stable without vasopressors and was transferred to a normal care unit. The affected finger developed necrosis and had to be amputated (Figure 1(b)).

**Figure 3:**
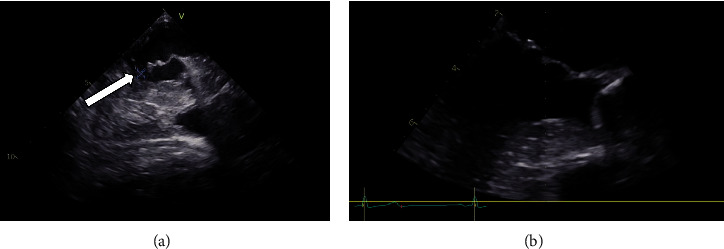
(a) Endocarditic lesion (4 × 5 mm) on the anterior mitral valve leaflet in the transesophageal view and (b) transesophageal echocardiographic control 8 weeks after diagnosis of endocarditis without detection of vegetation on the mitral valve.

Because of the initially reduced ejection fraction, a transthoracic echocardiogram was performed which showed an irregular structure on the mitral valve. A transesophageal ultrasound was performed and a diagnosis of mitral valve endocarditis of the anterior leaflet was made, and the size of the vegetation was 4 × 5 mm (Figure 3(a)). Because of the small size of the vegetation and the clinical presentation of the patient, it was treated conservatively with meropenem.

A kidney biopsy was performed because of suspected TMA and showed diffuse acute tubular damage with single-cell necrosis. There was no evidence of renal involvement of TMA. The kidney function did recover 2.5 months after admission, and dialysis was no longer required.

The patient was discharged 4 weeks after the bite, mainly because she refused prolonged inpatient anti-infective therapy. An outpatient dialysis therapy three times a week with administration of ertapenem was performed, which was later switched to oral cefpodoxime by the treating outpatient physician. Four weeks after the initial ultrasound, the vegetation was regressive. Eight weeks after the initial echocardiography, the endocarditis vegetations were no longer detectable (Figure 3(b)).

## 3. Discussion

Mark Twain once wrote “It's not the size of the dog in the fight, it's the size of the fight in the dog.” In this case, it was the presence of *Capnocytophaga canimorsus* in his mouth rather than the size of the dachshund which led to the life-threatening infection.

*C. canimorsus* is an encapsulated Gram-negative facultative anaerobe rod of the family Flavobacteriaceae and is found in the oral cavity of dogs and more rarely in cats [8]. It was first described by Bobo and Newton in a case report of septicemia with meningitis in 1976 [9] and since then been reported to cause a variety of pathologies including septicemia, meningitis, endocarditis, or purpura fulminans [10]. Mortality ranges between 10% and 30% in ICU cohorts [1]. Infection usually occurs through bites or saliva from cats or dogs. Transmission of *Capnocytophaga canimorsus* from person to person has not yet been described in the literature. We recommend asking about the presence of any pets in the household in cases of unclear sepsis in immunosuppressed patients.

After a bite, the incubation period until the appearance of the first symptoms is about 5 days [11]. Risk factors for the occurrence of a serious infection are immunosuppression (functional), asplenia, or alcohol abuse [12]. Our patient reported to drink one to two alcoholic beverages (approx. 20 g ethanol/d) per day; otherwise, she had no known immunodeficiency.

TTP and DIC have been described in connection with sepsis caused by *Capnocytophaga canimorsus* [13, 14]. In this case, a diagnosis of hemolysis and detection of schistocytes could not be made. The DIC score was negative with only two points for thrombocytopenia. Because of normal ADAMTS13 antibodies and ADAMTS13 antigen, TTP was ruled out.

TMA is characterized by endothelial damage formation of platelet thrombi in small arterial and venous vessels and abnormalities in the endothelium and vessel wall. The vessel lumen may close as a result and lead to terminal organ damage [15]. Hematologic findings include microangiopathic hemolytic anemia (MAHA) and thrombocytopenia. MAHA is due to mechanical damage of red blood cells, and laboratory chemistry reveals schistocytes. The thrombocytopenia results from platelet consumption during formation of the microthrombi. In patients with TTP, thrombocytopenia is usually severe [16].

Our patient was initially diagnosed with TMA. TTP was ruled out after normal levels of ADAMTS13 antibodies and antigen. Not all laboratory values for DIC have been obtained, but fibrinogen levels stayed in the normal range during the stay on the intensive care unit which could speak against the presence of DIC. On the other hand, fibrinogen is decreased in only a quarter of the patients with DIC and does not exclude the diagnosis of DIC [17].

Our patient only scored two points in the ISTH DIC score. For patients with sepsis, another scoring system, the Sepsis-Induced Coagulopathy (SIC) score, is more sensitive than the ISTH DIC scoring system. The score consists of three subitems, namely, INR, absolute platelet count, and SOFA score [18]. Applied to our case, our patient had a SIC score of four points and was therefore positive for SIC according to the score. We recommend using the SIC score in addition to the ISTH DIC score for patients with sepsis.

Our case presents a different differential diagnosis of the hemolytic anemia and thrombocytopenia. Renal involvement of TMA was not seen in the kidney biopsy, but the presence of schistocytes and thrombopenia indicate the diagnosis of TMA.

The persistent kidney injury could be seen as a complication of sepsis caused by *C. canimorsus*.

Endocarditis was an incidental finding during transthoracic echocardiography. There had been no abnormal heart sounds on the clinical examination, and the patient did not present with typical endocarditis-associated skin lesions. Endocarditis caused by *C. canimorsus* has been described in literature, but identification and resistance testing of this pathogen are challenging due to its fastidious growth pattern and its slow-growing nature [19]. Hence, the prolonged time to obtain accurate antimicrobial susceptibility testing results may hamper de-escalation of broad-spectrum antibiotics to a specific, targeted treatment (i.e., ampicillin–sulbactam).

Aortic and tricuspid valves are most commonly affected in *C. canimorsus* endocarditis [20, 21]. Our patient had a vegetation on her native mitral valve which has been described very rarely in literature [22]. In the cases reported, surgical repair of the mitral valve was necessary. In our patient, a conservative procedure was possible due to the preserved valve function.

## 4. Conclusion

The initially assumed TMA could not be confirmed in further investigations but was an important differential diagnosis in this case. *C. canimorsus* can present with a variety of clinical presentations and can suggest a TMA at first glance. The normal coagulation parameters and the nondecreased fibrinogen levels ensured that DIC was considered unlikely using the DIC score. We therefore see advantages in using the SIC score as a screening method in early stages of sepsis.

Mitral valve endocarditis is a rare complication of *C. canimorsus* sepsis, but our case shows it can be treated conservatively if the valve function is preserved.

Even minor injuries can cause life-threatening sepsis, so high-risk groups should be specially screened.

Given the variety of symptoms, complications and the high mortality, we recommend that patients with *C. canimorsus* sepsis be treated at centers with experience with this particular bacterium.

## Figures and Tables

**Table 1:** Laboratory values of the patient.

**Laboratory value**	**Standard value**	**d1**	**d2**	**d3**	**d4**	**d5**
Hemoglobin (g/dL)	12.0–16.0	11.9	9.9	10.1	7.9	7.4
Leukocytes (10^9^/L)	3.6–10.5	5.4	21.1	17.6	15.7	12.5
Platelets (10^9^/L)	140–400	114	30	39^∗^	5	7
IPF (%)	1.1–6.1		11.2	5.4	32.9	21.5
Creatinine (mg/dL)	0.5–0.9	1.41	2.83	4.05	2.93	2.1
Urea (mg/dL)	17–48	42	71	110	79	40
CRP (mg/L)	<5.0	186.2	259.4	303.3	70.1	38.4
PCT (ng/mL)	<0.05		118	148	86.3	
Interleukin-6 (pg/mL)	<7		330	106	49.4	
PTT (sec.)	23–31	30	55	40	27	23
Fibrinogen (mg/dL)	180–400	451	342	419	309	
LDH (U/L)	<289		579	1083	573	
Schistocytes (per mL)				20		
Haptoglobin (mg/dL)	30–200		74	<5		
Free hemoglobin (mg/dL)	<50		60	40		

^∗^Platelets increased after transfusion of one platelet concentrate.

## Data Availability

The datasets used and/or analyzed during the current study are available from the corresponding author on reasonable request. All data sharing statements are subject to conformity with German data protection legislation and rules (Datenschutzgrundverordnung [DGSVO]).

## Nomenclature

ADAMTS13: A disintegrin-like metalloproteinase with a thrombospondin type 1 motif, member 13
aHUS: Atypical hemolytic uremic syndrome
CRP: C-reactive protein
DIC: Disseminated intravascular coagulation
ELISA: Enzyme-linked immunosorbent assay
HIT: Heparin-induced thrombopenia
HUS: Hemolytic uremic syndrome
IPF: Immature platelet fraction
LDH: Lactate dehydrogenase
MAHA: Microangiopathic hemolytic anemia
PCT: Procalcitonin
SIC: Sepsis-induced coagulopathy
SOFA: Sepsis-Related Organ Failure Assessment score
TMA: Thrombotic microangiopathy
TTP: Thrombotic thrombocytopenic purpura
vWF: von Willebrand factor
